# Diffuse Alopecia and Thyroid Atrophy in Sheep

**DOI:** 10.3390/ani11123530

**Published:** 2021-12-10

**Authors:** Rubia Avlade Guedes Sampaio, Franklin Riet-Correa, Francisca Maria Sousa Barbosa, Daniela Dantas de Gois, Raquel Costa Lima, Iara Geovana da Silva, Vitória Maria da Silva, Alexandra Melo Oliveira, Sara Vilar Dantas Simões, Ricardo Barbosa Lucena

**Affiliations:** 1 Graduate Program in Animal Science, Universidade Federal da Paraíba, Areia 58397-000, PB, Brazil; rubia_avlade@yahoo.com.br (R.A.G.S.); franklinamaral@ufba.br (F.R.-C.); francistrabalhos@gmail.com (F.M.S.B.); lexa.mello@gmail.com (A.M.O.); saravdsimoes@gmail.com (S.V.D.S.); 2 Graduate Program in Animal Science in the Tropics, Universidade Federal da Bahia, Salvador 40170-110, BA, Brazil; 3 Laboratory of Veterinary Pathology, Universidade Federal da Paraíba, Areia 58397-000, PB, Brazil; danieladantas.dg@gmail.com (D.D.d.G.); raquel-lim@hotmail.com (R.C.L.); iarageovanas14@gmail.com (I.G.d.S.); vms3@academico.ufpb.br (V.M.d.S.)

**Keywords:** dermatoses, hypothyroidism, endocrine disease, mineral deficiency, ruminants

## Abstract

**Simple Summary:**

Thyroid dysfunction substantially affects quality of life, causing disturbances in different organs. A low intake of selenium and zinc can predispose to thyroid changes, resulting in hypothyroidism. A deficiency of selenium and zinc causes thyroid dysfunction and skin lesions. This paper describes natural cases of diffuse alopecia and thyroid abnormalities in sheep with selenium and zinc deficiency. The sheep had severe alopecia, and the thyroid hormones serum concentrations were below the reference values for the species. Zinc and Se concentrations were low in the serum and liver, and the thyroid gland was smaller than normal size. The present study is important as it is the first study examining zinc and Se deficiencies causing hypothyroidism in sheep. Furthermore, thyroid disorders affect the quality of life of humans and animals, and are associated with many organ-specific and non-organ-specific disorders.

**Abstract:**

Thyroid dysfunction substantially affects the quality of life due to its association with various disorders in different organs. A low intake of selenium and zinc can predispose to thyroid alterations, resulting in hypothyroidism. A deficiency of selenium and zinc causes direct and indirect skin lesions, both by the action of free radicals on the skin and by thyroid dysfunction. The aim of this study was to describe natural cases of diffuse alopecia and thyroid abnormalities in sheep with selenium and zinc deficiency. Five adult sheep presented marked and diffuse alopecia, and the residual hairs were dry and brittle. The skin was thick and crusty, with marked peeling. The triiodothyronine (T3) and thyroxine (T4) serum concentrations were below reference values for the species. Zinc and Se concentrations were low in both the serum and liver. During necropsy, cachexia associated with serous fat atrophy was observed, and the thyroid glands showed marked atrophy. Microscopically, the thyroid presented multifocal to coalescent atrophy, with atrophied and dilated follicles, macrophage infiltration, and the presence of fibrous connective tissue. The skin revealed hyperkeratosis and edema. It is concluded that thyroid atrophy, alopecia, and hyperkeratosis are associated with low serum and liver concentrations of zinc and selenium in sheep.

## 1. Introduction

Thyroid hormones activate the nuclear transcription of many genes in practically all cells, influencing the functional activity of the entire organism [1]. Approximately 93% of secreted thyroid hormones consist of thyroxine (T4) and 7% are triiodothyronine (T3). However, all thyroxine is eventually converted by deiodinases into triiodothyronine in tissues, so both are functionally important [2].

Thyroid hormone production may be affected by different factors, including mineral deficiencies such selenium [3], as these microelements are important for thyroid gland homeostasis [4,5]. Selenium is stored in the thyroid and is incorporated into selenoproteins (glutathione peroxidases, thioredoxin reductases, and iodothyronine deiodinases), which protect the gland from oxidative injuries during hormone production [4]. The deficiency of this mineral decreases the function of important selenoproteins, in particular iodothyronine deiodinases, which are responsible for the conversion of T4 to T3 [6]. The decrease of T3 results in stimulating the hypothalamic–pituitary axis to secrete thyroid-stimulating hormone (TSH) [7]. This rise in TSH typically stimulates the iodothyronine deiodinases to higher conversion T4 to T3, increasing the hydrogen peroxide (H_2_O_2_), which is not adequately removed by less active glutathione peroxidase selenoprotein [7]. Leakage of the H_2_O_2_ produces oxidative stress and damage to thyrocytes, with subsequent fibrosis [4,7].

Recent studies suggest that zinc is also involved in the regulation of deiodinase activity, thyroid releasing hormone, and TSH (thyrotrophin) synthesis [4,5]. This mineral acts as an inhibitor or cofactor of deiodinase enzymes (type 1 and type 2 deiodinases) [7]. Zinc also acts as a cofactor for deiodination reactions, which turn T4 into T3 [5]. In an experimental model, zinc deficiency induces atrophy of the thyroid, depletion of T3 and T4, and systemic effects [8]. 

Thyroid hormones, zinc, and selenium also influence epidermal homeostasis. Thyroid hormones interfere directly with the biology of the epidermis, dermis, and hair due to direct effects on skin receptors in epidermal keratinocytes, fibroblasts, dermis muscles, sebaceous glands, vascular endothelial cells, Schwann cells, and various types of cells that compound the hair follicle [9]. Zinc is essential for the catalytic, structural, and regulatory functions of proteins and/or enzymes involved in skin morphogenesis, and defense and repair processes [10,11,12]. Selenium is present as part of thioredoxin reductase and glutathione peroxidase, which share a major role in cell defense against oxidative stress in the skin [10]. 

Among all endocrinopathies, thyroid disorders are not well known in farm animals [13]. To our knowledge, the interaction between selenium and zinc deficiencies in the development of sheep hypothyroidism and skin changes has not been studied. This study aimed to describe natural cases of diffuse alopecia and thyroid alterations in sheep with low concentrations of selenium and zinc in the serum and liver.

## 2. Materials and Methods

### 2.1. Animal Ethics

The study was approved by the Animal Use Ethics Committee of the Federal University of Paraíba (approval no. 6983140418).

### 2.2. Animal Population Studied

Between 2016 and 2018, a total of 150 sheep were submitted to necropsy. Among these, five sheep Santa Inês and crossbred Dorper × Santa Inês (three ewes and two rams) had a history of diffuse skin disease. The sheep were of the Santa Inês breed and were crossbred from Santa Inês and Dorper, belonging to three farms (Farm 1, Farm 2, and Farm 3) located in Areia (Farm 1 and 2) and Remigio (Farm 3) in the Brejo region of the state of Paraíba, Brazil. Due to the seasonal variability of precipitation in the semiarid region, within each year, there are two distinct periods—a wet period from February to August and a dry period from September to January. During the dry period, the native forage is insufficient to sustain small ruminant feed quantity and quality needs; thus, it is necessary to establish a plan for supplementation [14].

### 2.3. Determination of Serum Thyroid Hormone Levels and Serum and Hepatic Mineral Concentrations

Serum concentrations of total triiodothyronine (T3) and total thyroxine (T4) hormones were measured by chemiluminescence assay (Roche Diagnostics^®^, Basel, Switzerland) on the Roche cobas e411 analyzer [15].

Fresh liver samples collected during the necropsies of five sheep were fragmented and taken to a forced ventilation oven for 72 h for pre-drying. Subsequently, the samples were placed in a watch glass and dried in an oven at 103 °C for 24 h to obtain the dry matter. Liver (0.2 g) and serum (1 mL) samples were placed in Teflon^®^ (PTFE) tubes and 12 mL diacid mixture (HNO_3_:HCl, 3:9) was added [16] using a CEM MDS-2000 microwave digestion (CEM Corporation, Charlotte, NC, USA). For the determination of minerals, the samples were diluted in deionized (Milli-Q^®^) water (Merck KGaA, Hesse, Darmstadt, Germany) [17]. The flame-emission spectroscopy with the nitrous oxide–acetylene flame method on the Optima^tm^ 2000 ICP optical emission spectrometer (PerkinElmer^®^ ICP-OES, Santa Clara, CA, USA) was used to determine the serum concentrations of cobalt (Co), copper (Cu), iron (Fe), molybdenum (Mo), selenium (Se), and zinc (Zn). This technique was also used to determine the concentrations of Cu, Fe, Se, and Zn in the liver.

### 2.4. Pathology

The five sheep died spontaneously, and the samples were collected during necropsy from the skin of the limbs, trunk, and head; the thyroid gland; all internal organs; the brain; and bones (femur, rib, and vertebrae). A representative sample of 250 g of the liver was refrigerated, in order to determine the mineral levels. The samples of the other part of the liver and the other organs were placed in 10% buffered formaldehyde, fixed for 48 h, routinely processed in paraffin-embedded alcohols and xylols, and cut at 4 µm. The slides were stained with hematoxylin and eosin (HE) for histopathological analysis in Olympus BX 45 microscope (Olympus Corporation, Tokyo, Japan).

## 3. Results

### 3.1. Clinical Evaluation 

The animals were between three and five years old. They were raised with extensive grazing in the Caatinga, a semi-arid region. In wet and dry parts of the year, the feeding of sheep is based on pasture. The animals did not receive roughage or concentrate feeding in the trough and mineral supplementation, except for iodized salt (25 µg/g of salt) ad libitum in the troughs. The main complaints were hair loss and progressive weight loss. The cases always occurred from September to November, during the dry period. Sheep deaths were reported from flocks, affecting only adult sheep—on one farm, these deaths affected 10% (1 out of 10); on another, 30% (3 out of 10); and on another, 40% (3 out of 12). All of the affected animals died.

**Figure 1:**
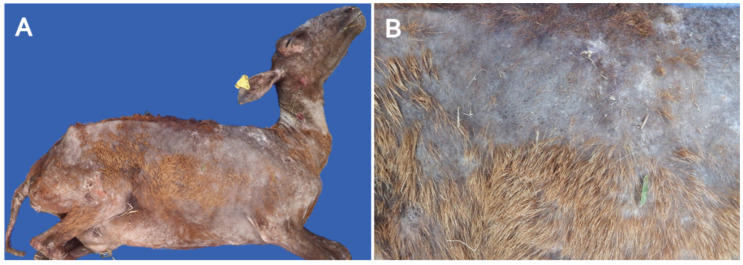
Diffuse alopecia associated with thyroid disorder in adult sheep. (**A**) Diffuse alopecia in adult sheep. (**B**) Alopecia, hyperkeratosis, and hyperpigmentation of the skin.

General physical examination revealed apathy, weight loss, hypothermia, weakness, and bradycardia. Dermatological evaluation of the five adult sheep (three females and two male sheep) identified diffuse alopecia (Figure 1A). The few areas of the skin that were not alopecic had dry and brittle hair. The skin was thickened, crusted, and markedly peeling (Figure 1B). 

### 3.2. Serum Concentrations of Thyroid Hormones and Minerals

The T3 and T4 concentrations were below the reference values for the species (Table 1). The serum and liver zinc and selenium concentrations were low. The serum and hepatic concentrations of copper, cobalt, and iron were within normal ranges (Table 2 and Table 3).

### 3.3. Pathologic Alterations

During necropsy, the main findings were cachexia, serous fat atrophy, and marked thyroid gland atrophy. Additional changes included periodontitis and mandibular abscess in a crossbred ram, liver abscess in a crossbred ewe, and endometritis and placental retention in a Santa Inês ewe.

**Figure 2:**
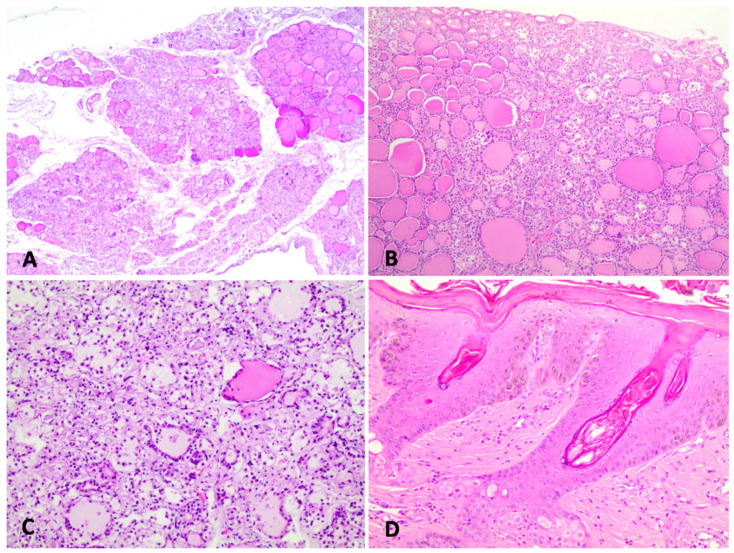
Thyroid and skin micrographs of sheep. (**A**) The architecture of the thyroid gland is markedly altered due to inflammatory infiltration of lymphocytes and replacement of follicles with fibrous connective tissue. HE, obj. 10×. (**B**) Follicles with an initial colloid degeneration process are noted. HE, obj. 20×. (**C**) There is marked inflammation and destruction of thyroid follicles. HE, obj. 20×. (**D**) Severe epidermal and follicular hyperkeratosis. HE, obj. 20×.

Histopathological evaluation of the thyroid revealed multifocal to coalescent atrophy (Figure 2A), characterized by small follicles lined by flattened cells, with little or no colloid inside. On the other hand, some follicles were dilated (Figure 2B). In some areas, there was rupture of the follicle wall, with macrophage infiltration (Figure 2C). In other areas, there was abundant fibrous connective tissue surrounding the atrophic follicles. Microscopic evaluation of the skin revealed hyperkeratosis and marked acanthosis in the epidermis and inside the follicles (Figure 2D).

## 4. Discussion

The results show that diffuse alopecia and thyroid atrophy cases indicate trace mineral deficiency in small ruminant flocks in the Semiarid Northeast Brazil region. The role of selenium and zinc in the maintenance of normal thyroid function is well known in the literature [7,21,22,23]. However, to our knowledge, thyroid atrophy associated with simultaneous deficiency of selenium and zinc is reported for the first time in the present study. Thus, it might suggest that the identified hypothyroidism in sheep was primary, which occurs when the thyroid tissue cannot produce its hormones properly [24]. 

Signs of apathy, hypothermia, bradycardia, and skin alterations identified in animals are associated with hypothyroidism. Thyroid hormones influence the metabolic activity of many tissues [25]. Thus, a decreased metabolic rate leads to hypothermia and cold intolerance [26]. Bradycardia is related to the fact that the myocardium is the tissue that has the majority of the body’s thyroid hormone receptors, which affects the frequency and duration of the action potential of cardiac myocytes [27]. Low T3 levels reduce the rate of systolic depolarization and diastolic repolarization, and increase the duration of the action potential and refractory period of the atrioventricular node [28]. Regarding skin changes, in vitro studies have suggested that keratinocytes with T3 depletion have decreased levels of the plasminogen activator, an enzyme implicated in the loss of corneocytes [29], resulting in hyperkeratosis, a lesion observed in all five sheep affected by thyroid atrophy.

Analysis of the serum and hepatic mineral concentrations showed a significant reduction in selenium and zinc concentrations, with zinc deficiency being more pronounced, as zinc concentrations were even more than 50% lower than those found normally in the species [20]. Deficiency of these micronutrients can result in severe damage to different organs and tissues, especially the thyroid gland and skin [9,30,31,32]. The results of the mineral concentrations indicate a simultaneous selenium and zinc deficiency in sheep.

The thyroid gland is characterized as a tissue with a high selenium concentration, being the organ with the highest amount of selenium per gram of tissue [6]. Selenium depletion can compromise the action of selenoproteins, which remove oxygen free radicals generated during the production of thyroid hormones. Atrophic and fibrotic histological changes identified in the thyroid indicated that the gland had been affected by a chronic process, suggesting that the tissue suffered slow oxidative damage, as these minerals act in organ defense against radicals [10,33].

The histopathologic changes observed in the sheep were similar to the experimental zinc deficiency in guinea pigs [8]. Both species had fibrosis and severe thyroid gland atrophy, characterized by degenerated follicles, associated with mononuclear inflammatory cell infiltrate. In another experimental study, the administration of zinc in the diet of rats causes an increase in the concentration of thyroid hormone (T4 and T3) levels and induces a significant reduction in the level of TSH [30]. It is known that this mineral is a part of and modulates the function of structural proteins [10]. In humans, the relationship between zinc and thyroid function is increasingly evident. People with low concentrations of this mineral, when given zinc in their diet along with thyroxine treatment, a considerable improvement in mental depression was noted, as well as the regression of skin lesions and hair loss and improved appetite and taste [34].

The changes identified in the skin are also associated with the effect of selenium and zinc deficiencies in the skin. Zinc is mainly concentrated in the basal cells of the epidermis [35] where proteins that store this element have antioxidant properties, called metallothioneins (MTs). Deficiency of this element results in MT dysfunction and alterations in the proliferation and maturation of the keratinized epithelium [31]. Zinc deficiency in sheep and goats causes diffuse alopecia, thickening, and marked hyperkeratosis and/or parakeratosis in the epidermis and interior of the follicles [36,37,38], which are similar to the lesions observed in the sheep in this research. However, these studies did not report thyroid lesions. Selenium, in turn, is present in the skin as part of glutathione peroxidase and thioredoxin reductase, which participate in the cellular defense against oxidative stress [10,33].

The severe disease observed in the sheep suggests that the interaction between zinc and selenium deficiency results in more serious injuries than in cases where these deficiencies occur in isolation. Zinc deficiency not associated with selenium deficiency causes diffuse alopecia in goats, called zinc-responsive dermatopathy [37]. In goats, this deficiency is limited to a dermatological condition, and this disease shows a good response to supplementation [36]. In our study, the animals were not supplemented.

In addition to skin lesions caused by mineral deficiency, dermatopathy can be aggravated by a deficiency of thyroid hormones. Thyroid hormones act on epidermal homeostasis, and in sheep, mineral deficiencies and hypothyroidism are interrelated conditions [9]. Studies have shown that zinc deficiency can decrease the amount of T3 in animal and human plasma [39,40], and that supplementation with this mineral can elevate thyroid hormones [40]. In contrast, thyroid failure may further compromise zinc deficiency, as its hormones are essential for the absorption of this mineral. In other words, primary zinc failure will compromise thyroid function, and thyroid failure will secondarily impair the absorption of this mineral [34,41,42].

The deficiencies may result from deficits in pastures, resulting from low concentrations in the soil (primary deficiency), or from interactions between minerals that modify the soil absorption (secondary deficiency). Zinc deficiency, for example, may result from competition with other nutrients, such as copper and iron [43]. However, despite low selenium and zinc concentrations, the serum concentrations of Cu, Mo, Co, and Fe and the liver concentrations of Cu were within normal values for the species, suggesting the occurrence of primary selenium and zinc deficiency in the region of study. The cases always occurred during the dry period when the availability and quality of forage are compromised [44]. Mineral supplementation, mainly zinc, is indicated during the dry period. Zinc bioavailability in ruminants appear to be affected by dietary factors that have not yet been clearly defined [45]. 

In the present study, iodine concentrations were not evaluated. However, sheep had free access (ad libitum) to iodized salt (table salt) in the trough. In addition, all kinds of table salt contains iodine, as iodine fortification of table salt is mandatory in Brazil [46]. This mineral is an essential constituent of thyroid hormones. Sheep flocks with low levels of iodine develop abortion and goiter. Adult animals are rarely clinically affected, although they do suffer infertility, deterioration in semen quality and loss of libido in the male, poor wool growth, depressed milk yield, and reduced weight gain [47]. Reproductive problems or goiter were not observed in our study. Furthermore, thyroid atrophic was observed in all sheep, unlike the colloid goiter in iodine deficiency disorders, characterized by a marked hypertrophy and hyperplasia of the cuboidal epithelium of the thyroid follicles, with little or no colloid production, and follicular collapse [47]. Future studies are needed to investigate the concentrations of minerals (i.e., selenium, zinc, copper, molybdenum, and iodine) in sheep flocks, mainly to identify subclinical cases of mineral deficiency.

The differential diagnosis for sheep with diffuse alopecia includes poisoning by the plant *Leucaena leucocephala*, characterized by weight loss, ulcerations of the tongue and esophagus, sialorrhea, goiter, infertility, or death [48,49]. These clinical signs result from the action of mimosine [50], which prevents iodine peroxidation in the thyroid (iodination), with a decreased synthesis of T3 and T4 [51]. However, leucaena was not cultivated on farms, and the sheep in this study never ingested this plant.

## 5. Conclusions

Our study showed that alopecic and hyperkeratotic dermatopathy, thyroid atrophy, systemic weakness, and high mortality in sheep in Northeast Brazil are associated with low concentrations of selenium and zinc in the serum and liver and with hypothyroidism. The investigation and development of appropriate control and prevention measures of pasture and soil should be conducted.

## Figures and Tables

**Table 1:** Thyroid hormone, triiodothyronine (T3) and thyroxine (T4), concentrations in sheep with a history of diffuse alopecia and thyroid atrophy.

Hormone (nmol/L)	Sheep					Normal [13,18,19]
	1	2	3	4	5	
T3	2.23	NA	2.01	2.05	2.03	2.04–5.85
T4	40.58	NA	40.55	45.08	41.03	49.68–146.46

NA: not evaluated.

**Table 2:** Serum mineral concentrations in sheep with diffuse alopecia and thyroid atrophy.

Minerals	Sheep					Normal [20]
	1	2	3	4	5	
Cobalt (ng/mL)	1.1	NA	1.4	1.2	0.7	0.18–2.0
Copper (µg/mL)	0.8	NA	0.99	1.3	0.8	0.75–1.7
Iron (µg/mL)	1.03	NA	1.35	1.43	1.55	0.9–2.7
Molybdenum (ng/mL)	3.04	NA	3.9	3.04	3.65	1.0–5.0
Selenium (µg/mL)	0.04	NA	0.05	0.02	0.025	0.06–0.2
Zinc (µg/mL)	0.35	NA	0.30	0.20	0.18	0.55–1.2

NA: not evaluated.

**Table 3:** Mineral concentrations in the liver (dry weight) of sheep with diffuse alopecia and thyroid atrophy submitted to necropsy.

Minerals (mg/kg)	Sheep					Normal [13,20,21]
	1	2	3	4	5	
Copper	168	187	194	233	124	135–500
Iron	288	302	204	244	344	181–380
Selenium	NA	0.3	NA	NA	NA	0.8–3.0
Zinc	34.6	55.4	60.0	32.1	29.8	101–200

NA: not evaluated.
